# Seasonal dynamics in taxonomy and function within bacterial and viral metagenomic assemblages recovered from a freshwater agricultural pond

**DOI:** 10.1186/s40793-020-00365-8

**Published:** 2020-10-28

**Authors:** Jessica Chopyk, Daniel J. Nasko, Sarah Allard, Anthony Bui, Mihai Pop, Emmanuel F. Mongodin, Amy R. Sapkota

**Affiliations:** 1 Maryland Institute for Applied Environmental Health, University of Maryland School of Public Health, College Park, MD USA; 2 Department of Pathology University of California San Diego, La Jolla, California USA; 3 Center for Bioinformatics and Computational Biology, Institute for Advanced Computer Sciences, University of Maryland, College Park, MD USA; 4 Institute for Genome Sciences and Department of Microbiology and Immunology, University of Maryland School of Medicine, Baltimore, MD USA

**Keywords:** Metagenome, Shotgun, Agricultural irrigation, Antibiotic resistance, Microbial communities, Bacteria, Bacteriophage

## Abstract

**Background:**

Ponds are important freshwater habitats that support both human and environmental activities. However, relative to their larger counterparts (e.g. rivers, lakes), ponds are understudied, especially with regard to their microbial communities. Our study aimed to fill this knowledge gap by using culture-independent, high-throughput sequencing to assess the dynamics, taxonomy, and functionality of bacterial and viral communities in a freshwater agricultural pond.

**Results:**

Water samples (*n* = 14) were collected from a Mid-Atlantic agricultural pond between June 2017 and May 2018 and filtered sequentially through 1 and 0.2 μm filter membranes. Total DNA was then extracted from each filter, pooled, and subjected to 16S rRNA gene and shotgun sequencing on the Illumina HiSeq 2500 platform. Additionally, on eight occasions water filtrates were processed for viral metagenomes (viromes) using chemical concentration and then shotgun sequenced. A ubiquitous freshwater phylum, *Proteobacteria* was abundant at all sampling dates throughout the year. However, environmental characteristics appeared to drive the structure of the community. For instance, the abundance of *Cyanobacteria* (e.g. *Nostoc*) increased with rising water temperatures, while a storm event appeared to trigger an increase in overall bacterial diversity, as well as the relative abundance of *Bacteroidetes.* This event was also associated with an increase in the number of antibiotic resistance genes. The viral fractions were dominated by dsDNA of the order *Caudovirales*, namely *Siphoviridae* and *Myovirdae.*

**Conclusions:**

Overall, this study provides one of the largest datasets on pond water microbial ecology to date, revealing seasonal trends in the microbial taxonomic composition and functional potential.

## Background

Ponds are small (1 m^2^ to ~ 50,000 m^2^), shallow, standing water bodies that are found ubiquitously among Earth’s terrestrial biomes, with an estimated 2.6 to 9 million ponds located within the U.S. alone [1, 2]. Globally, ponds occupy a greater total area than lakes and are considered to be functionally and ecologically distinct, playing a major role in the global cycling of carbon and supporting a high level of macro- and micro- species diversity [1–6]. Along with those that are formed by natural processes, there are many ponds that are human constructed for a variety of recreational, industrial, agricultural, and aesthetic purposes [1, 7]. For instance, in areas where municipal and ground water sources are limited or unavailable, ponds are built to capture and store water for irrigation [8, 9]. Despite the importance of ponds to both environmental and human activities, the majority of research on freshwater resources is focused on large water systems (e.g. lakes). As a result, outside of extreme environments (e.g. saline/hypersaline [10–12], thermokarsts [13]), and aquaculture facilities [14–16] ponds remain largely understudied [17], especially with regard to their microbial communities.

Microbial communities are vital to the health and maintenance of aquatic ecosystems [18]. However, due to their topography (e.g. size small and shallow depth) ponds are uniquely sensitive to anthropogenic and environmental factors [19]. Nonpoint source nutrient pollution, coupled with warm temperatures, and long water residence times can result in a high abundance of algal and cyanobacterial concentrations, in some cases leading to blooms that deplete oxygen levels and produce toxins [20–23]. Storm events can also trigger an influx of fecal pathogens that can contaminate irrigation supplies and subsequently crops [24–26]. For instance, a 2002 multistate outbreak of *Salmonella* Newport on tomatoes was traced back to contaminated pond water used for irrigation [27]. In addition to pathogens, runoff can introduce pollutants originating from land use practices (e.g. antibiotics, pesticides) [28]. Because of the long water retention times of ponds, these pollutants may then diffuse and accumulate, leading in some cases to changes in bacterial community dynamics, including increased selection pressures for antibiotic-resistant bacterial populations [29]. However, the persistence of these disruptions and foreign bacterial agents depends on complex factors such as sedimentation, temperature, UV light, and predation [30].

Despite the value in surveying the microbial composition of ponds, the limited collection of previous studies have been largely restricted to PCR or culture-based methodologies and often comprise just a static “snap shot” of the microbial community. Thus, we are restricted in our understanding of microbial functionality, dynamics, and response under multiple conditions. Shotgun metagenomics makes it possible to observe and analyze a broad sampling of microbial diversity without cultivation, providing new insights into their genomic complexity and functional potential [31]. In addition, because shotgun metagenomic sequencing does not rely on a universally distributed marker gene, such as the 16S rRNA gene, it can also be used to explore the viral community [32].

Using metagenomics previous studies have identified that within the viral community of surface freshwater sites bacteriophages (phage) dominate [33–37]. Phages are critical components in shaping the evolution, diversity, abundance, and genetic composition of bacteria [38]. Temperate phages (forming prophage) can influence their hosts’ phenotype through the horizontal transfer of genes, such as those for antibiotic resistance/toxins and those that promote host fitness and adaptability [39, 40]. However, phage composition, diversity, and host-interactions are often linked to fluctuating environmental characteristics [41]. Therefore, assessing phage ecology and relationships with their host(s) is critical with regard to completing a comprehensive characterization of pond biodiversity.

In the present study, we periodically sampled surface water from a freshwater agricultural pond located in the Mid Atlantic, United States. From these samples, we employed culture-independent high-throughput sequencing to characterize the dynamics, taxonomy, and functionality of their bacterial and viral communities over time.

## Materials and methods

### Study site and sample collection

Water samples (total *n* = 14) were collected on the following dates: 6/12/17, 7/17/17, 8/8/17, 8/21/17, 9/11/17, 9/25/17, 10/30/17, 11/13/17, 12/18/17, 1/22/18, 2/12/18, 3/12/18, 4/9/18, and 5/7/18 from a freshwater agricultural pond located in a rural area of central Maryland, United States (maximum depth of ca. 3.35 m and a surface area of ca. 0.26 ha). At each date, a utility transfer pump (0.08 W; Everbilt, Atlanta, GA) powered by a EU1000i generator (American Honda Motor Co., Ltd., Alpharetta, GA) and connected to a sampling cartridge via vinyl braided tubing (1.9 cm inner diameter, Sioux Chief, Peculiar, MO) was submerged 15–30 cm below the surface and used to pump roughly 10 L of water into a sterile polypropylene carboy. Samples were kept in the dark at 4 °C and processed within 24 h of collection.

### Water physicochemical assessment

At each time point a ProDSS digital sampling system (YSI, Yellow Springs, OH, United States) was used to measure the following physicochemical properties of the pond water: temperature (°C), pH, dissolved oxygen (% DO), conductivity (SPC uS/cm), oxidation-reduction potential (ORP, mv), turbidity (FNU), nitrate (mg/L), and chloride (mg/L). Using the Nation Weather Services historical data archive, ambient temperature was recorded for the time and date at each sampling event.

### Water sample processing

Microbial DNA was isolated as described in detail previously [6]. Briefly, for each sample 10 L of water was filtered sequentially through a Whatman 1 μm polycarbonate filter (Sigma-Aldrich, MO, United States) and a 142-mm diameter 0.2 μm membrane filter (Pall Gelman Sciences, MI, United States) attached via sterile 1.6 mm PVC tubing with a Watson Marlow 323 Series Peristaltic Pump (Watson-Marlow, Falmouth, Cornwall, United Kingdom).

The 1 μm polycarbonate filter was used initially to collect large cells and debris to make the subsequent 0.2 μm membrane filtration more effective and efficient. Following filtration, filters (1 and 0.2 μm) containing the cellular fraction were dissected into four equal quadrants and stored at − 80 °C until DNA extraction.

### Viral concentration and DNA extraction

On 6/12/17, 7/17/17, 8/8/17, 8/21/17, 9/11/17, 9/25/17, 10/30/17, and 5/7/18 the iron chloride procedure was used on the pond water after sequential 1 μm and 0.2 μm filtration, with the 0.2 μm a common pore size used in virome generation to prevent cellular contamination [33, 37, 42]. A 1 mL solution of FeCl_3_ (4.83 g FeCl_3_ into 100 ml H_2_O) was added to the filtered pond water and incubated in the dark for 1 h. The samples were then filtered onto 142-mm 1 μm polycarbonate filters (Sigma-Aldrich, MO, United States) to capture flocculated viral particles [43]. Filters were stored at 4 °C in the dark until resuspension. For resuspension, filters were rocked overnight at 4 °C in 10 mL of 0.1 M EDTA - 0.2 M MgCl_2−_ 0.2 M Ascorbate Buffer, described in detail elsewhere [43]. Resuspended viral particles were then subjected to a DNase I (Sigma-Aldrich, MO, United States) treatment for 1 h and passed through a 33-mm diameter sterile syringe filter with a 0.2 μm pore size (Millipore Corporation, MA, United States). DNA was extracted from 500 μl of the viral concentrate using the AllPrep PowerViral DNA/RNA Kit (Qiagen, CA, United States) per the manufacturer’s instructions. Prior to sequencing, viral DNA was tested for the presence of bacterial contamination via 16S rRNA gene PCR.

### Microbial DNA extraction

Microbial DNA was extracted from the filters using an enzymatic and mechanical lysis procedure [6, 44]. Each filter quadrant was placed in a lysing matrix tube with a cocktail of PBS buffer, lysozyme, lysostaphin, and mutanolysin. After incubation at 37 °C for 30 min, a second lysing cocktail (Proteinase K and SDS) was added followed by another incubation at 55 °C for 45 min and mechanical lysis via bead beating with a FastPrep Instrument FP-24 (MP Biomedicals, CA) (6.0 m/s for 40s). The resulting DNA was purified with the QIAmp DNA mini kit (Qiagen, CA, USA) and assessed for quality with the NanoDrop 2000 Spectrophotometer. To create a composite sample, microbial DNA extracts from all four quadrants of both filter sizes were pooled for each date.

### 16S rRNA gene sequencing and analysis

From each of the pooled microbial DNA extractions (*n* = 14), the V3-V4 hypervariable region of the 16S rRNA gene was PCR-amplified and sequenced on the Illumina HiSeq 2500 (Illumina, San Diego, CA, United States) utilizing a dual-indexing strategy for multiplexed sequencing developed at the Institute for Genome Sciences [45].

The resulting 16S rRNA reads were screened for low quality bases and short read lengths, merged with PANDAseq, de-multiplexed, and trimmed of artificial barcodes and primers [46–48]. Using VSEARCH, reads were then checked for chimeras with the UCHIME algorithm and the ChimeraSlayer RDPGold_Trainset reference training dataset [49]. Chimera-free reads were then clustered de novo into Operational Taxonomic Units (OTUs) using VSEARCH with a minimum confidence threshold of 0.97. Following OTU clustering, alpha diversity (Observed OTUs) was calculated and assessed using the R packages: Bioconductor [50], metagenomeSeq [51], vegan [52], phyloseq [53], fossil [54], biomformat [53], and ggplot2 [55] on unrarefied data and data rarefied to an even sampling depth (13,956 sequences). Taxonomic assignments via 16S rRNA were not considered in this study.

### Shotgun sequencing for microbial metagenomes and viromes

For both the microbial (*n* = 14) and viral (*n* = 8) samples, DNA extracts were shotgun sequenced. Briefly, for each sample DNA was used in a tagmentation reaction, followed by 12 cycles of PCR amplification using Nextera i7 & i5 index primers per the modified Nextera XT protocol. The final libraries were then quantitated by Quant-iT hsDNA kit. The libraries were pooled, loaded onto an Agilent High Sensitivity D1000 ScreenTape System, and then sequenced on an Illumina 2500 Hiseq X10 flow cell (Illumina, San Diego, CA, United States) targeting 100 bp paired-end reads per sample.

### Microbial and viral metagenomic assembly

After sequencing the paired-end reads from both microbial and viral libraries were quality trimmed using Trimmomatic ver. 0.36 (sliding window:4:30 min len:60) [56]. The quality reads were then merged with FLASh ver. 1.2.11 [57] and assembled de novo with MEGAHIT [58]. Open reading frames (ORFs) were predicted and translated from each library using MetaGene [59].

### Microbial and viral taxonomic and functional classification

For the microbial metagenomes, translated peptide ORFs were searched against UniRef 100 (retrieved May 2018) via protein-protein BLAST (BLASTp ver. 2.6.0+) (E value ≤1e-3) [60, 61]. Max cumulative bit score was used to assign taxonomy and calculated by summing the bit scores of all taxa with a hit to a translate peptide ORFs encoded by the contig. Translated peptide ORFs were also searched against the SEED database using BLASTp (E value ≤1e-3). Translated peptide ORFs were assigned to a SEED subsystem with the maximum sum bit score among all of the ORF’s hits. Taxonomic and functional classification of viromes were conducted as described in Chopyk et al., 2017 [6].

For both viral and microbial metagenomes coverage was calculated for each contig by recruiting quality-controlled reads to assembled contigs via Bowtie2 ver. 2.3.3 in very sensitive local mode. Then using the “depth” function in Samtools ver. 1.4.1 we computed the per-contig coverage [62]. Coverage for translated peptide ORFs were denoted by start and stop coordinates within each contig. To normalize abundances across libraries, contig and ORF coverages were divided by the sum of coverage per million, similar to the transcripts per million (TPM) metric used in RNA-Seq [63, 64]. Scripts performing these assignments and normalization are available at https://github.com/dnasko/baby_virome. All taxonomic and functional data were visualized using the R packages ggplot2 ver. 3.1.0 and pheatmap ver 1.0.10 [55, 65].

### ARGs prediction and host assignment

Translated peptide ORFs from both viral and microbial metagenomes were searched against the “Comprehensive Antibiotic Resistance Database” (CARD; retrieved July 2018) via protein-protein BLAST (BLASTp ver. 2.6.0+) (E value ≤1e-3) [60, 66]. A queried translated peptide ORF was considered an ARG if it had > 40% coverage and > 80% amino-acid identity to a CARD protein [67, 68]. In addition, for the ARGs conferring resistance through target mutations, a post-processing step was taken to confirm the presence of resistance-conferring mutations; a MAFFT alignment with reference sequences available at CARD [69]. Taxonomic assignments were parsed for contigs containing ARG-like translated peptide ORFs. Networks were visualized by Cytoscape [70].

### Statistical analysis

Significance tests were conducted using an ANOVA with post hoc Tukey’s HSD Test among meteorological seasons, defined by the American Meteorological Society. Additionally, to identify associations between the water physicochemical characteristics and the normalized abundance of the bacterial genera, as well as between the abundance of bacterial genera and viral families, Pearson’s correlation coefficients were calculated in RStudio version 1.0.153 and corrected for multiple comparisons with FDR.

## Results

### Sequencing effort and assembly

All samples (*n* = 22) were sequenced on the Illumina HiSeq, 14 microbial and eight viral. In total, there were 907,056,944 read pairs for the microbial metagenomes with an average of 64,789,782 read pairs per metagenome (+/− 7,936,115 Standard Deviation, SD) (Table S1). For the viral metagenomes, there were 489,222,408 read pairs with an average of 61,152,801 read pairs per metagenome (+/− 9,064,079 SD) (Table S2). After assembly, there were a total of 9,979,705 contigs generated, with an average of 712,836 contigs per sample (+/− 142,125 SD) for the microbial metagenomes and a total of 1,913,254 contigs, with an average of 239,157 contigs per sample (+/− 45,658 SD) for the viromes.

### Temporal variations in physicochemical characteristics and bacterial diversity of pond water

**Fig. 1:**
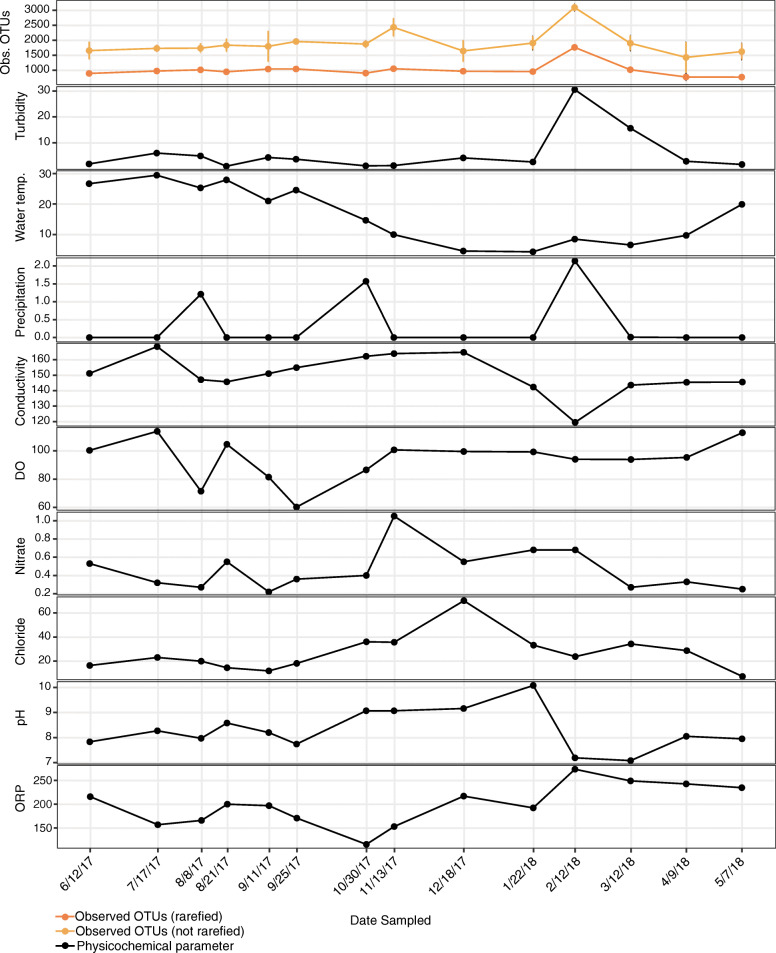
Temporal dynamics of the physicochemical properties and bacterial diversity in agricultural pond water**.** Line graph displaying the alpha diversity (Observed OTUs, rarefied; dark orange, raw; light orange) and physicochemical properties (black) through time. The following physicochemical properties were surveyed: temperature (°C), pH, dissolved oxygen (% DO), conductivity (SPC uS/cm), oxidation-reduction potential (ORP mv), turbidity (FNU), nitrate (mg/L), and chloride (mg/L). Sampling dates are ordered temporally

Physicochemical variables for each sampling date are shown in Fig. 1. Water temperature ranged from 29 °C (7/17/17) to 4 °C (1/22/18). By meteorological season, winter (12/18/17, 1/22/18, 2/12/18) had an average water temperature of 6 °C. This was significantly (*p* ≤ 0.05) lower than autumn (9/11/17, 9/25/17, 10/30/17, 11/13/17) and summer (6/12/17, 7/17/17, 8/8/17, 8/21/17), which had an average water temperature of 18 °C and 27 °C, respectively. In addition, the water temperature in summer was significantly (*p* ≤ 0.05) higher than spring (3/12/18, 4/9/18, 5/7/18). The only other environmental factor that significantly (*p* ≤ 0.05) differed by meteorological season was ORP, which was significantly higher in spring compared to autumn. Precipitation 24-h prior to sampling occurred only on 8/8/17, 10/30/17, and 2/12/18.

Furthermore, we examined the bacterial diversity at each time point by way of amplification and sequencing of the 16S rRNA gene. Overall, the alpha diversity, surveyed by rarefied and unrarefied Observed OTUs, was generally steady throughout the year, with no significant differences found between rarefied or unrarefied diversity and meteorological season. However, by physicochemical parameter we did find some correlations. Specifically, we found that turbidity was significantly (*p* ≤ 0.05) positively correlated with the abundance of rarefied and unrarefied Observed OTUs.

### Temporal variations in bacterial phyla of pond water

For the microbial metagenomes collected throughout the year, on average 78% (+/− 4% SD) of contigs could be assigned a taxonomic representative (Table S3). Of these, the majority was homologous to Bacteria 93% (+/− 2% SD), followed by Eukaryota 3% (+/− 1% SD), and Viruses 3% (+/− 0.5% SD).

**Fig. 2:**
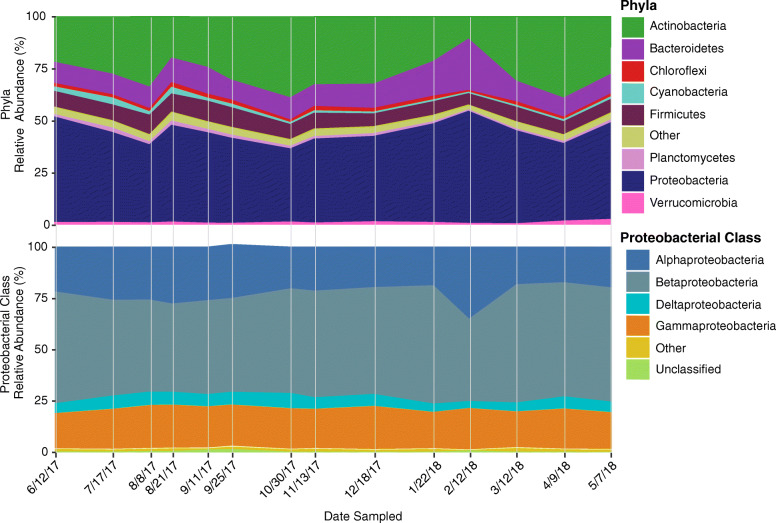
Temporal dynamics of bacterial composition in agricultural pond water. Stacked area chart depicting the composition of bacterial communities at the phylum level, as well as with the *Proteobacteria* phylum split into classes. Sampling dates are ordered temporally. Normalized abundance measured as contig coverage divided by the sum contig coverage per million and presented as a percentage

For each of the contigs, a normalized abundance was calculated to account for assembly proficiency and sequencing depth and then parsed for those assigned as Bacteria. Of these, the most frequently observed bacterial phylum was *Proteobacteria*, which accounted for 43% (+/− 5%) of the total bacterial assigned abundance across all time points (Fig. 2). The next most abundant phyla were *Actinobacteria* at 28% (+/− 8%), *Bacteroidetes* at 12% (+/− 4% SD), and *Firmicutes* at 7% (+/− 1%). The largest phylum, *Proteobacteria,* was composed chiefly of the class *Betaproteobacteria* 50% (+/− 6% SD) and *Alphaproteobacteria* 23% (+/− 5% SD), with the largest spike in *Alphaproteobacteria* occurring on 2/12/18.

By meteorological season, winter had a significantly (*p* ≤ 0.05) higher abundance of *Bacteroidetes* than all other seasons, while summer had a significantly (*p* ≤ 0.05) higher abundance of *Cyanobacteria* compared to all other seasons. Summer and autumn both had a high abundance of *Firmicutes*, with both significantly (*p* ≤ 0.05) higher than spring and winter.

In addition to differences by meteorological season, the normalized abundance of some of these top phyla correlated with physicochemical parameters surveyed in the water: *Actinobacteria* (R = 0.65, *p* ≤ 0.05) correlated with conductivity, *Bacteroidetes* correlated with precipitation (R = 0.63, *p* ≤ 0.05), conductivity (R = -0.67, *p* ≤ 0.05), and turbidity (R = 0.74, *p* ≤ 0.05), and *Chloroflexi* correlated negatively with precipitation (R = -0.68, *p* ≤ 0.05) and turbidity (R = -0.63, *p* ≤ 0.05). Additionally, *Cyanobacteria* (R = 0.83, *p* ≤ 0.01), *Firmicutes* (R = 0.80, *p* ≤ 0.01), and *Planctomycetes* (R = 0.74, *p* ≤ 0.01) all correlated positively with water temperature.

### Temporal variations in bacterial genera of pond water

**Fig. 3:**
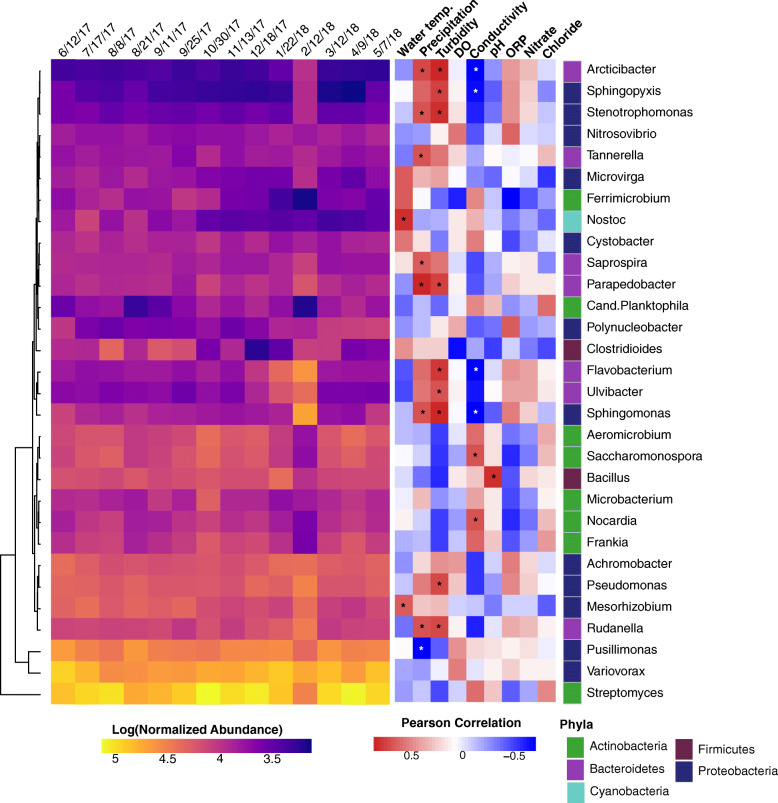
Bacterial genera and correlations with physicochemical factors in agricultural pond water across sampling dates. Heatmaps based on the log-transformed normalized abundance of the most dominant genera (> 1% in at least one sample) and the Pearson’s correlation coefficients between the water physicochemical factors and the bacterial genera normalized abundance listed on the Y-axis. Genera annotated with colors representative of their phylum (*Proteobacteria*: dark blue, *Actinobacteria*: green, *Firmicutes*: burgundy, *Bacteriodetes*: purple, *Cyanobacteria*: light blue). Hierarchical clustering of genera was performed using the complete clustering method with Euclidean distances on the bacterial abundances. Sampling dates ordered temporally. Asterix denote significant Pearson correlations (*p* ≤ 0.05) after FDR correction. Normalized abundance measured as contig coverage divided by the sum contig coverage per million

Within the bacterial assignments classified at the genera level, *Streptomyces* (11% +/− 3% SD), *Variovorax* (7% +/− 2% SD), *Pusillimonas* (4% +/− 1% SD), and *Pseudomonas* (3% +/− 0.5% SD) were the most abundant (Fig. 3). By meteorological season, winter had a significantly (*p* ≤ 0.05) higher abundance of *Ulvibacter*, *Rudanella*, and *Flavobacterium* compared to all seasons. Spring had a significantly (*p* ≤ 0.05) higher abundance of *Polynucleobacter* compared to all seasons and a significantly higher abundance of *Nitrosovibrio* compared to autumn. Summer had a significantly (*p* ≤ 0.05) higher abundance of *Nostoc* compared to all seasons. Autumn had a significantly (*p* ≤ 0.05) higher abundance of *Ferrimicrobium* compared to winter.

Similar to the analysis of the bacterial phyla, we calculated Pearson’s correlations between the normalized abundance of the dominant bacterial genera and the physicochemical parameters of the pond water (Fig. 3). In total, precipitation and turbidity correlated with the greatest number of genera, followed by conductivity and water temperature.

### Microbial metagenome, functional potential

**Fig. 4:**
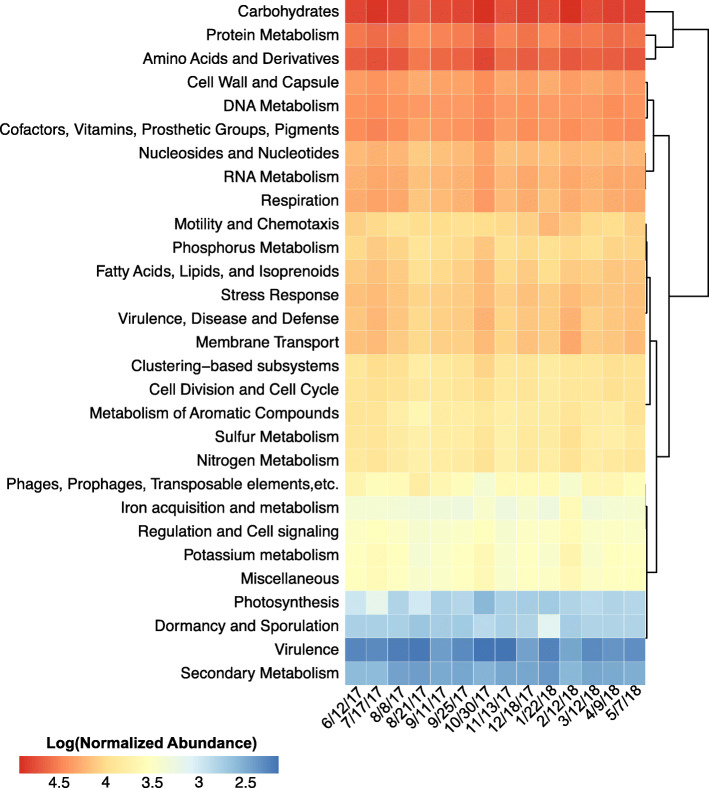
Functional composition in agricultural pond water across sampling dates. Heatmap of the microbial metagenomes’ functional profiles, represented by the SEED systems, at each sampling date. Hierarchical clustering of SEED systems was performed using the complete clustering method with Euclidean distances. Sampling dates are ordered temporally. Normalized abundance measured as ORF coverage divided by the sum ORF coverage per million

On average, 40% (+/− 3%) of translated peptide ORFs from the microbial metagenomes could be assigned a SEED functional category (Fig. 4). Of these, “Carbohydrate Metabolism” was the most abundant representing on average 16% (+/− 1%) of the total assigned functional abundance followed by “Amino Acids and Derivatives” at 12% (+/− 0.3%), “Protein Metabolism” at 9% (+/− 0.4%), and either “Cofactors, Vitamins, Prosthetic Groups, Pigments” at 7% (+/− 0.2%) or “DNA Metabolism” at 6% (+/− 0.5%). By meteorological season, the only SEED functional category that was significantly (*p* ≤ 0.05) different among seasons was “Motility and Chemotaxis”, which was significantly higher in winter compared to autumn and summer.

Similar to the bacterial abundance, precipitation was significantly correlated with the abundance of a diversity of functional SEED systems including: “Potassium metabolism” (R = 0.78, *p* ≤ 0.001), “Regulation and Cell signaling” (R = 0.76, *p* ≤ 0.01), “Iron acquisition and metabolism” (R = 0.74, *p* ≤ 0.01), “Virulence Disease and Defense” (R = 0.72, *p* ≤ 0.01), “Miscellaneous” (R = 0.69, *p* ≤ 0.01), “Phages Prophages Transposable elements etc.” (R = -0.69, *p* ≤ 0.05), “Carbohydrates” (R = 0.68, *p* ≤ 0.05), and “Membrane Transport” (R = 0.67, *p* ≤ 0.01). Likewise, turbidity was also correlated with “Iron acquisition and metabolism” (R = 0.76, *p* ≤ 0.01).

### Antibiotic resistance and host taxonomy in pond water

**Fig. 5:**
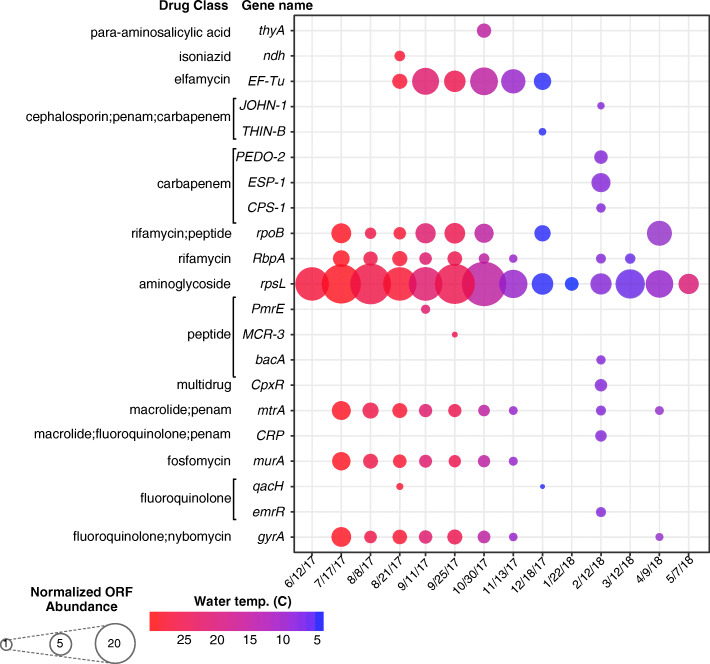
Antibiotic resistance genes (ARGs) in agricultural pond water across sampling dates. Dot plot of the ARG-like translated peptide ORFs predicted from the microbial metagenomes at each sampling date. The size of each dot is equivalent to the normalized ORF abundance with homology to each ARG listed on the y-axis, and the color representative of the temperature of the water at the time of sampling. Normalized abundance measured as ORF coverage divided by the sum ORF coverage per million

To assess antibiotic resistance in the microbial and viral metagenomes, we conducted a BLAST analysis of translated peptide ORFs against CARD. No translated peptide ORFs within the viral metagenomes had significant homology to ARGs within CARD. However, in the microbial metagenomes, 184 translated peptide ORFs were identified as 21 unique ARGs conferring resistance to over 15 drug classes (Fig. 5). For the ARGs whose resistance is associated with target mutations, they were confirmed to carry the following mutations: *rpsL*; K88R [71]; *gyrA*, S95T [72]; *murA* C117D [73]; *rpoB* H526T [74]; *EF-Tu* Q124K [75]; *ndh* V300G, V246A [76]. A normalized abundance was also calculated for each ARG-like translated peptide ORF. From this, the greatest abundance of ARG-like translated peptide ORFs was attributed to the sampled collected on 10/30/17, followed by 9/25/17 and 9/11/17. However, the greatest diversity of ARGs was identified on 2/12/18.

**Fig. 6:**
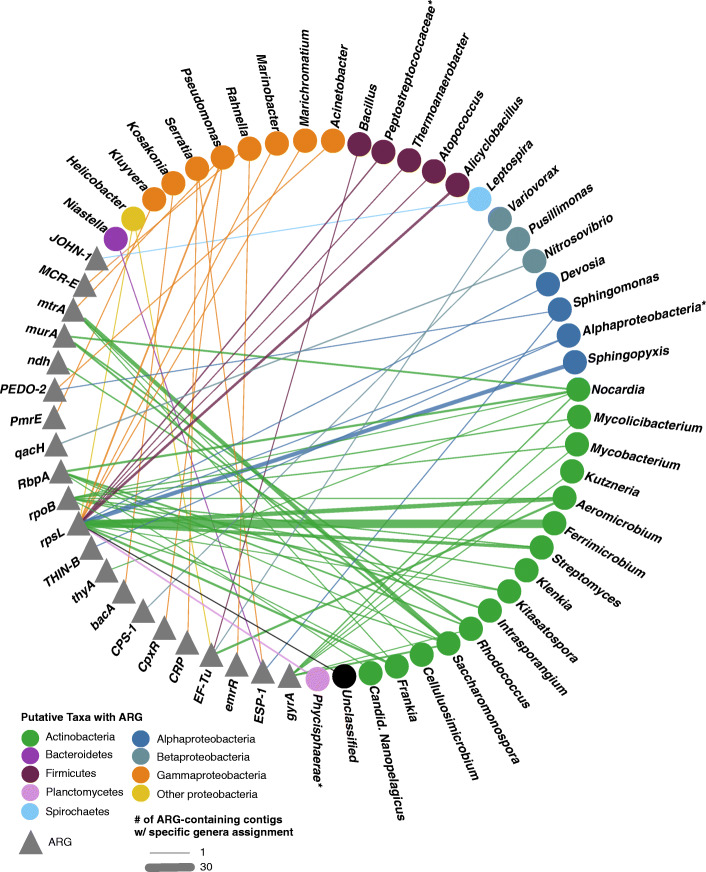
ARG host network. Bipartite network of the bacterial taxa with predicted antibiotic resistance genes (ARGs). Grey triangles represent ARGs connected by an edge to its putative bacterial host, with each edge colored by the host phyla. Bacterial host defined as the taxa assigned to the contig the ARG-like translated peptide ORF originated from and colored accordingly. Asterix represent taxa that could not be assigned at the genus level

For each ARG-like translated peptide ORF, the source genus and phylum were parsed (Fig. 6). All the ARG-like translated peptide ORFs originated from contigs assigned as Bacteria. Of these, 71% of were contigs assigned to the phylum *Actinobacteria* (9 unique ARGs), largely of the genus *Ferrimicrobium* (30 *rpsL*), *Saccharomonospora* (1 *RbpA*, 4 *gyrA*, 12 *mtrA*, 4 *murA*, 2 *rpsL*), and *Aeromicrobium* (5 *EF-Tu*, 1 *rpoB*, 13 *rpsL*). The next largest phylum assigned to contigs with an ARG-like ORF was *Proteobacteria*, which accounted for 21% of the contigs, but had a wide diversity of ARGs (14 unique ARGs). Within this phylum, *Sphingopyxis* (12 *rpsL)* and *Pseudomonas* (3 *rpsL*, 1 *CpxR*, 1 *mtrA*) were assigned to the most contigs.

### Viral taxonomic and functional composition

**Fig. 7:**
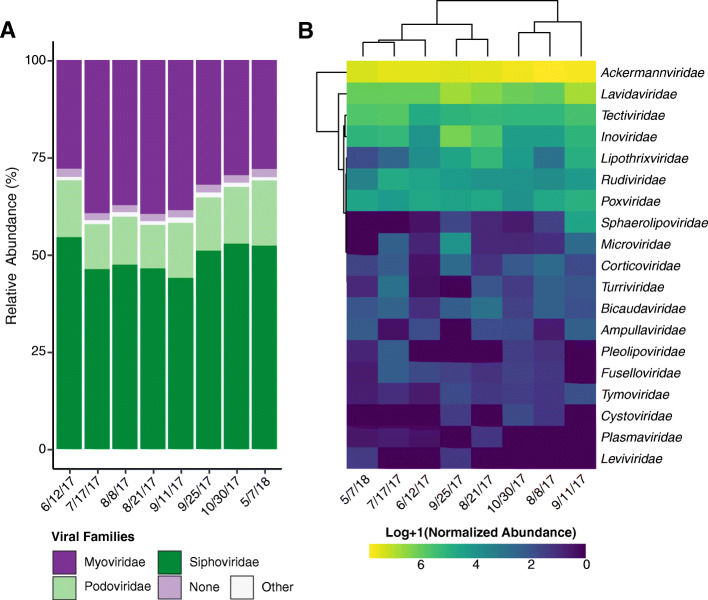
Viral composition in agricultural pond water across sampling dates. (**a**) Stacked bar charts depicting the composition of the viral communities at the family level. Normalized abundance measured as contig coverage divided by the sum contig coverage per million and presented as a percentage. (**b**) Heatmap based on the (log+ 1)-transformed normalized abundance of “other” viral families. Hierarchical clustering of samples and viral taxa were performed using the complete clustering method with Euclidean distances

For the viromes, on average 47% of contigs (+/− 1%) could be assigned a taxa, which is in agreement with results described in other viral metagenomic studies [77]. For those that could be assigned, a normalized abundance was calculated. The vast majority of viral abundance was assigned to the tailed bacteriophage of the order *Caudovirales* (Fig. 7). Of these, the majority were similar to members of the *Siphoviridae* (49% +/− 4%) family, followed by the *Myoviridae* (34% +/− 5%) and *Podoviridae* families (14% +/− 2%). The remaining portion were either viral contigs that could not be assigned a family (2% +/− 0.1%) or were other viral families (1% +/− 0.2%). The other viral families included viruses infecting other bacteria and archaea, ssDNA bacteriophage *Microviridae* and *Inoviridae*, plant viruses from the family *Tymoviridae*, and animal/arthropod viruses from the family *Poxviridae.*

On average, only 10% (+/− 2%) of translated peptide ORFs from the viromes could be assigned at a SEED system (Fig. S1). Of these, “DNA Metabolism” was the most abundant representing on average 23% (+/− 5%) of the total assigned functional abundance followed by “Phages, Prophages, Transposable elements” at 12% (+/− 0.3%), “Motility and Chemotaxis” at 9% (+/− 1%), and “Protein Metabolism” at 7% (+/− 1%). For the dominant viral phyla we did not calculate any significant correlations between their relative abundance or functional potential and the physicochemical properties of the pond water, likely due to the limited sample size (*n* = 8).

## Discussion

Freshwater is a finite natural resource essential to life on Earth. It is critical in supporting urban, agricultural, and industrial activities, as well as providing a home for a rich diversity of macro- and micro- organisms [1–6]. Yet, anthropogenic activities, climate change, and a growing global population threaten its quality and availability worldwide [78, 79]. Here, we focused our attention on one freshwater resource, ponds, that have been historically disregarded in favor of studies on larger aquatic systems.

In this study, the pond freshwater was dominated by *Proteobacteria*, largely that of *Betaproteobacteria*, a class found ubiquitously in freshwater [80]. This agrees with initial freshwater samples collected along Indian Pond in New York U.S., as well as a recent study from our lab surveying the microbial composition in a freshwater creek [81, 82]. However, here we were able to detect seasonal changes in the abundance of the bacterial phyla that corresponded to environmental conditions. For instance, during the summer months, the abundance of *Cyanobacteria*, increased with increasing ambient water temperature (Figs. 2 and 4). This is not surprising as water temperature has been found in multiple prior studies to be predictors of the abundance of *Cyanobacteria* [83, 84]. Moreover, these results agree with those reported in an earlier study from our lab, where we found, through 16S rRNA gene sequencing, that the relative abundance of *Cyanobacteria* and *Synechococcus* decreased significantly with declining temperature [6]. In this study, *Cyanobacteria* peaked in the summer season (specifically on 7/17/17), but continued at high abundance into autumn, where mild temperatures likely sustained their growth. During these peak seasons, the genus *Nostoc* was the most abundant within the *Cyanobacteria* phylum (Fig. 3).

The *Nostoc* genus includes a highly diverse range of nitrogen-fixing species, commonly found in aquatic environments as either free-living, engaged in cooperative growth on plants and fungi, or in gelatinous colonies on rocks and stones [85]. While *Nostoc* blooms in freshwater ponds and lakes are often just considered a nuisance, there are still concerns with regard to recreational use or agricultural irrigation post and during a bloom [86, 87]. *Nostoc* spp. are becoming increasingly recognized for their role in the production of cyanotoxins, as well as other bioactive compounds that can cause serious health problems in humans and animals [86, 87]. In fact, *Nostoc* is reported by the EPA as one of the eight most common microcystin-producing *Cyanobacteria* [88]. In humans, microcystin exposure is associated with both acute health effects (e.g. abdominal pain, headache, diarrhea, pneumonia, etc.) and chronic conditions (e.g. primary liver cancer, colon and rectum carcinomas) [89, 90]. While we do not know from the data presented in this study if the *Nostoc* spp. are toxin-producing, their persistence in the summer months is cause for future investigation to protect environmental and public health.

In addition to fluctuations driven by seasonal trends, we saw a large shift in the bacterial composition that correlated with a sizable precipitation event on 2/12/18 (Fig. 1). Likely, this event triggered an influx of upland runoff into the pond, resulting in an increase in bacterial diversity, as well as an increase in the abundance of *Bacteroidetes* (e.g. *Rudanella, Flavobacterium*) and *Proteobacteria* (e.g. *Alphaproteobacteria*) (Fig. 2). *Bacteroidetes* are often limited in freshwater environments, likely due to their dependency on organic matter [80, 91]. However, previous studies have found *Bacteroidetes* increased in abundance within freshwater creeks following storm events [92, 93]. In these studies, the authors suggested that the increase in *Bacteroidetes* may be a concern, as they are often indicative of human fecal and sewage material contamination [94, 95]. In fact, they have been suggested as better alternatives to traditional fecal indicators such as *E. coli* or fecal coliforms [94–96]. Along with potential pathogens and a diversity of terrestrial microorganisms, runoff can also introduce upland pollutants, such as antibiotics.

While antibiotics and ARGs are both naturally occurring, nonpoint and point source pollution of human and animal-derived wastes may select for an abundance that is atypical and may ultimately have repercussions for environmental and public health [97, 98]. Freshwater environments have become established as important reservoirs for the potential maintenance and dissemination of ARGs, especially small lakes and pond [99]. These lentic bodies tend to have longer water retention times compared to lotic environments, which can result in the accumulation of antibiotics and selection for resistant bacteria [100, 101]. In this study, we identified ARGs on all of the sampling dates conferring resistance through a wide range of mechanisms across clinical, veterinary, and agricultural antibiotics. This varied resistome may be attributed to the selective forces driven by the pond topography, environmental contributions, and the commensal bacterial community composition. Unlike other surface freshwater sites, the pond surveyed here was dominated by *Streptomyces* of the phylum *Actinobacteria*. *Actinobacteria*, particularly *Streptomyces*, produce many clinically significant antibiotics [97, 102, 103]. As a result, they can contain a wide array of ARGs for self-protection, as well as those inherited horizontally from other *Actinobacteria* [104, 105]. Thus, it was not surprising to see that the majority of ARG putative hosts originated from *Actinobacteria* (Fig. 6).

As for the environmental contributions, the largest spike in ARG diversity was on 2/12/18, which corresponded to a large precipitation event. Here, we saw the emergence of seven unique ARGs (*JOHN-1*, *ESP-1*, *CRP*, *PEDO-2*, *CPS-1*, *CpxR*, and *bacA*) conferring resistance to a broad range of clinically- relevant antibiotics, including three beta-lactamases against carbapenem. The majority of these ARGs, unlike in the other months, were identified on contigs assigned as *Gammaproteobacteria.* This is consistent with the idea that these ARGs were introduced by an influx of upland runoff, as *Gammaproteobacteria* are not common in freshwater and are thought to be transient members introduced from the surrounding environment [80].

While other studies on freshwater have identified phage-encoded ARGs we did not observe any ARGs in the viral fraction [106, 107]. However, we did identify other putative functions carried largely by bacteriophages, specifically, genes related to DNA metabolism (Fig. S1). Previous studies characterizing DNA viruses in freshwater [108], wastewater treatment plants [109], and reclaimed water [110] found DNA metabolism genes were also enriched. This high abundance of virome-associated metabolic genes suggests elevated metabolic activity within these water systems. It also highlights the potential phages in these systems have to interfere in the metabolism of their hosts [111]. This could be of particular importance within the pond surveyed here, as we also identified a large abundance of *Siphoviridae*, a family of largely temperate dsDNA bacteriophage. For phages, lysogeny is suggested to be advantageous when conditions are poor, such as during times of nutrient-starvation [112]. Whereas, the lytic lifestyle is suggested to dominate when the bacterial community is the most productive (e.g. summer) [113]. While we do not have viral data that spans the coldest months of the year, the abundance of *Siphoviridae* did decrease and the abundance of *Myoviridae*, a traditionally virulent phage family, did increase during the warmer months (7/17/17–9/11/17) surveyed. However, the dominance of phage lifestyle strategy may be more complex then previously thought, as not all studies find lysogeny to be prevalent only in times of low bacterial productivity [114]. For instance, the “piggy-back-the-winner” model was born from observations that showed lysogeny is more prevalent at higher host cell densities [115, 116]. In this study, the prevalence of lysogenic phages may also be due to the composition of the host taxa, as the dominant bacterial phyla of the pond, *Actinobacteria*, and *Proteobacteria*, have been previously reported in some environments to be ideal hosts for temperate phages [117].

In addition to their significance as a freshwater resource for human industrial and agricultural activities, ponds are also a “hot spot” of biodiversity that significantly contribute to global ecosystem health [118]. Here, we provide one of the largest datasets on pond water microbial ecology to date. We expect these data will serve to not only improve understanding of the factors that may contribute to the disruption of pond biodiversity but also further our knowledge regarding the potential microbial risks of using pond water for agricultural irrigation.

## Conclusions

Ponds represent a potential resource for freshwater, especially in agricultural settings. Here, we characterized the seasonal fluctuations in the microbial communities within one of these complex and often understudied water bodies. We found, through the use of shotgun metagenomics, that features of the bacterial community are strongly influenced by seasonal forces, including temperature, conductivity, precipitation, and turbidity. For instance, we noted that the abundance of *Cyanobacteria* (e.g. *Nostoc* spp), increased with rising ambient water temperature. In addition we characterized the functional potential of the bacterial fraction and identified 21 unique ARGs conferring resistance to over 15 drug classes, with the majority of hosts identified as members of the *Actinobacteria* phylum. Interestingly, we found that the diversity of ARGs, largely from *Gammaproteobacterial* hosts*,* spiked with a large precipitation event. Moreover, for a subset of samples we were able to characterize the viral communities, an often overlooked, but incredibly important, member of freshwater systems. From these data we found that *Siphoviridae* and *Myoviridae* dominated the pond, with the latter increasing during the warmer months surveyed. Taken together, these data showcase the range of compositional and functional variability within a freshwater pond over the course of a year.

## Supplementary information

**Additional file 1: Figure S1.** Functional composition in agricultural pond water viral fraction across sampling dates. **Table S1.** Descriptive sequencing statistics for microbial metagenomes. **Table S2.** Descriptive sequencing statistics for viromes. **Table S3.** Contig taxonomic assignments for microbial metagenomes.

## Data Availability

Metagenomic reads were submitted to NCBI’s Sequence Read Archive under the BioProject accession number PRJNA473136.

## Abbreviations

CARD: Comprehensive Antibiotic Resistance Database
ARG: Antibiotic Resistance Gene
ORF: Open Reading Frame
DO: Dissolved Oxygen
ORP: Oxidation-Reduction Potential
