# Supplementary material for: Seasonal dynamics in taxonomy and function within bacterial and viral metagenomic assemblages recovered from a freshwater agricultural pond
Source: Environ Microbiome. 2020 Oct 28;15:18. doi: 10.1186/s40793-020-00365-8 (PMC8067656; doi:10.1186/s40793-020-00365-8)
Supplement: Supplementary file 1 — Additional file 1: Figure S1. Functional composition in agricultural pond water viral fraction across sampling dates. Table S1. Descriptive sequencing statistics for microbial metagenomes. Table S2. Descriptive sequencing statistics for viromes. Table S3. Contig taxonomic assignments for microbial metagenomes. [file 40793_2020_365_MOESM1_ESM.pdf]

Supplemental Figures

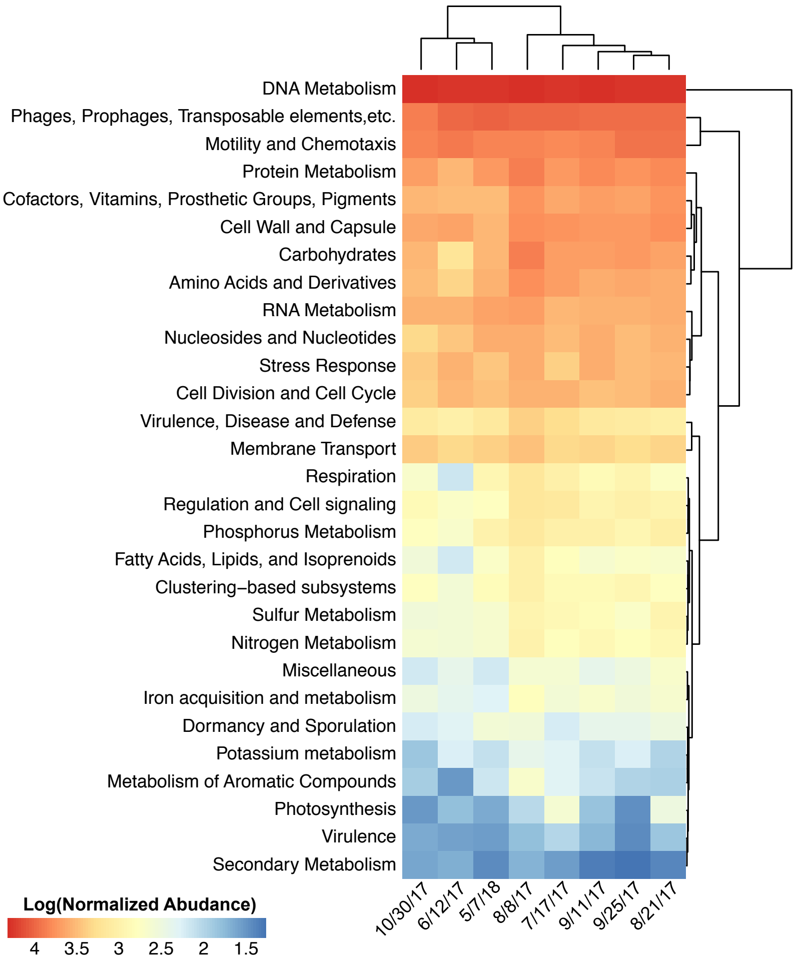

Figure S1: Functional composition in agricultural pond water viral fraction across sampling dates. Heatmap of the normalized abundance assigned to the SEED systems at each sampling date for the viromes. Hierarchical clustering of samples and SEED systems was performed using the complete clustering method with Euclidean distances. Normalized abundance measured as ORF coverage divided by the sum ORF coverage per million.

Supplemental Tables

Table S1: Descriptive sequencing statistics for microbial metagenomes

Date	No. Read Pairs	No. Contigs	Mean Contig Size	Median Contig Size	Max Contig Size	GC Content
6/12/17	56253234	710332	850	487	277707	49%
7/17/17	63779336	825414	807	477	117814	51%
8/8/17	64585947	785337	763	461	205041	49%
8/21/17	69809556	805980	843	486	322391	49%
9/11/17	64832833	815793	794	464	164272	49%
9/25/17	63781249	840001	777	466	227816	50%
10/30/17	59929487	665594	866	490	356886	48%
11/13/17	71006872	821949	831	476	500323	48%
12/18/17	69728632	759516	820	470	266589	48%
1/22/18	85726602	317644	681	441	133727	48%
2/12/18	56732600	762015	576	404	155163	49%
3/12/18	54167570	541845	799	453	456402	47%
4/9/18	65786989	651522	797	450	442516	46%
5/7/18	60936037	676763	868	473	443357	48%

Table S2: Descriptive sequencing statistics for viromes

Date	No. Read Pairs	No. Contigs	Mean Contig Size	Median Contig Size	Max Contig Size	GC Content
6/12/17	64110963	276729	865	470	180347	44.67%
7/17/17	62502798	273781	667	438	169626	46.72%
8/8/17	81168407	258993	729	452	226706	45.62%
8/21/17	56676868	254571	873	472	304756	44.08%
9/11/17	58609455	194360	704	451	163936	45.27%
9/25/17	56219160	153900	670	442	133962	46.72%
10/30/17	50692882	280625	715	454	89836	46.39%
5/7/18	59241875	220295	786	445	191403	45.58%

Table S3: Contig taxonomic assignments for microbial metagenomes.

Date	# Contigs Assigned Taxa	Contig Assignments			
		Bacteria	Archaea	Eukaryota	Virus
6/12/17	595070	558560	4446	10428	17018
7/17/17	682721	643284	5246	15071	13909
8/8/17	613967	575392	5086	13920	14510

8/21/17	638184	592345	6260	15298	18359
9/11/17	621912	580804	5118	14605	16113
9/25/17	647567	603594	5287	17045	16056
10/30/17	551826	520367	4081	11014	12117
11/13/17	601780	537650	4785	34534	18709
12/18/17	596552	550304	4303	19664	16942
1/22/18	259134	240201	1702	7537	7786
2/12/18	619155	588965	2679	15543	8704
3/12/18	402761	371034	2688	12175	13094
4/9/18	449222	409371	2896	20077	12875
5/7/18	499438	455695	3087	23363	13349
